# Multifidelity Topology Optimization with Runtime Verification and Acceptance Control: Benchmark Study in 2D and 3D

**DOI:** 10.3390/ma19040769

**Published:** 2026-02-16

**Authors:** Nikhil Tatke, Jarosław Kaczmarczyk

**Affiliations:** Department of Theoretical and Applied Mechanics, Faculty of Mechanical Engineering, Silesian University of Technology, Konarskiego 18A, 44-100 Gliwice, Poland; jaroslaw.kaczmarczyk@polsl.pl

**Keywords:** optimal shape, solid isotropic material with penalization, finite element analysis, preconditioned conjugate gradient solver, density filtering

## Abstract

Topology optimization using density-based approaches often requires high-resolution meshes to achieve reliable compliance evaluation and robustness against mesh dependency. However, increasing the problem sizes—especially in 3D—results in prohibitively expensive computation times. Coarse-mesh approaches significantly accelerate runtimes; however, they also introduce discretization errors that can guide the optimizer towards incorrect topology families if left unregulated. To address this issue, a multifidelity framework with acceptance control was developed that enables runtime verification and explicitly manages the optimizer state. The main idea is to use coarse discretizations to generate new design proposals and transfer candidate designs to fine discretizations at periodic intervals for verification. Proposals are then accepted or rejected using a best-referenced criterion; if verification fails, the optimizer reverts to the best verified state. The proposed framework balances fine-discretization accountability with coarse-discretization efficiency through configurable verification schedules and a cleanup phase. The framework is evaluated on standard 2D and 3D structural benchmark problems with deterministic load perturbations, and performance is assessed in terms of final verified compliance, wall-clock runtime, acceptance rate, and gray fraction.

## 1. Introduction

Topology optimization (TO) is a popular methodology for creating lightweight, high-performance structures in aerospace, automotive, and civil engineering applications [1,2]. Density-based formulations, such as solid isotropic material with penalization (SIMP), have been extensively used to enable the systematic distribution of materials within a design domain to minimize compliance under a volume constraint [3,4,5]. In addition to single objective SIMP, topology optimization also includes multi-criteria formulations using evolutionary algorithms [6,7], various other representations (e.g., level-set methods with implicit boundaries and phase-field methods with diffuse interfaces) [8,9,10,11]. This study focuses on SIMP-based compliance minimization to isolate the verification framework’s contribution, though the concept extends to other formulations.

Despite the maturity of these methods, the cost of computation increases rapidly with increasing problem size because each design iteration requires at least one large-scale linear solve, making 3-D applications much more expensive than 2-D benchmarks. For example, the typical 3-D cantilever discretization using 200 × 100 × 50 hexahedral elements results in approximately $3.1\times 10^{6}$ displacement DOFs; therefore, fine-mesh optimization can be expensive even with modern solvers and hardware acceleration [12,13,14].

One common way to accelerate optimization is to replace fine discretization with coarse discretization to reduce the number of DOFs and per-iteration cost. Coarse-mesh optimization provides substantial runtime savings but introduces a coarse–fine model mismatch. Discretization errors can drive the search toward designs that appear favorable on the coarse mesh but perform poorly at finer resolution [13,14,15,16,17]. This causes a practical trust problem: without some form of fine-mesh accountability, coarse-only optimizers may accept updates that are locally optimal to the coarse model but are detrimental to the fine model, and such degradation can be difficult to recover from once the optimizer’s state has progressed [18,19].

Although there are many examples of multifidelity optimization and model management [19,20] within the broader optimization community, relatively few apply directly to the specific case of density-based TO in a verification-aware manner, and systematic statistical validation across benchmarks remains limited [21].

This study introduces a runtime-verifiable multifidelity optimization framework with acceptance control for density-based topology optimization, referred to as the multifidelity acceptance safeguard (MFAS) framework. The main contributions of this paper are: (1) an explicit accept/reject mechanism based upon periodic fine-mesh verification of candidate designs with rollback to the best verified state. This provides fine-mesh accountability while utilizing the efficiency of coarse-mesh optimization; (2) a paired benchmark campaign of 60 runs (3 problems × 4 methods × 5 deterministic seeds) reporting compliance, runtime, acceptance rates, and gray fractions; and (3) a comparison of verification frequencies with a fine-mesh cleanup phase to investigate the cost–quality tradeoffs.

## 2. Related Work

In the following subsections, the density-based methods used in topology optimization literature are reviewed, followed by the state-of-the-art for accelerating the computation of the optimization process, and to provide the context for the multifidelity technique presented in this work.

### 2.1. Density-Based Topology Optimization

Topology optimization via a density-based methodology was first introduced by Bendsøe and Kikuchi [22], who represented the material distribution inside a given design domain using continuous density variables associated with each finite element. By adopting such a representation, the discrete problem of distributing materials is transformed into a continuous optimization problem solvable by the available gradient-based methods. One of the most popular schemes for interpolating density variables is the solid isotropic material with penalization (SIMP) method [3,23]. SIMP uses a power-law function relating element stiffness to density, penalizing intermediate values to push solutions toward binary (solid/void) configurations. The penalization factor (typically p=3 for compliance minimization) significantly affects optimizer behavior and convergence [3,12].

Regularization techniques ensure mesh-independent, manufacturable designs. To achieve these goals, two classes of regularization techniques have been developed: density filters [16] and sensitivity filters [24]. The former involves modifying the density values of the design variables before computing the stiffness matrix of the elements of the finite element mesh. In contrast, the latter modifies the gradients of the objective function and/or constraints before they are used by the optimizer. Both approaches eliminate checkerboard patterns and enforce a minimum feature size in the resulting designs. Recent quantitative studies have further highlighted the sensitivity of grayscale characteristics and resulting topologies to the chosen filter radius [25,26]. The choice of optimization algorithm significantly affects both solution quality and computational efficiency. A widely adopted optimization algorithm is the optimality criteria (OC) method [13,27]. Although it has the advantage of being relatively fast and easy to implement, it has several limitations, including a lack of generality with respect to the treatment of multiple constraints and objective functions. The method of moving asymptotes (MMA) handles arbitrary constraints at a higher computational cost [28]. Another class of algorithms is represented by the continuum-based optimality criteria (COC) [29], or convex linearization method (CONLIN), which lie between those of the OC and the MMA [30]. The aforementioned differences among the various algorithms produce different numbers of iterations, different convergence behaviors, and sensitivities to the initial guess, making it difficult to directly compare the performances of the optimization frameworks. Therefore, to correctly evaluate the performance of the proposed acceleration techniques, it is necessary to define common benchmarking conditions, that is, the same initial guess, filter parameters, convergence criterion, and mesh discretization.

### 2.2. Multifidelity Approaches

Multifidelity methods combine models with different costs and accuracy levels to accelerate applications such as uncertainty quantification, statistical inference, and optimization [19]. The fundamental premise is to leverage inexpensive low-fidelity models—such as coarse-mesh discretizations, reduced-order models, or simplified physics—to guide the solution process while maintaining accuracy through selective high-fidelity evaluations [19,20,31]. In topology optimization, a high-fidelity model typically corresponds to a fine-mesh finite element analysis, whereas low-fidelity models may employ coarser discretizations or data-driven surrogates.

The success of multifidelity optimization depends critically on the model management strategy, which determines how the work is distributed among the models while providing theoretical guarantees on accuracy and convergence. Model management approaches can be categorized into three types: adaptation, fusion, and filtering [19]. Adaptation strategies enhance low-fidelity models with information from high-fidelity evaluations as optimization proceeds. Fusion approaches combine outputs from multiple fidelity levels, as demonstrated by co-kriging methods that construct unified surrogate models from both coarse and fine evaluations [32,33]. Filtering methods screen candidates on low-fidelity models first, eliminating rejected designs from costly high-fidelity analysis [19].

Trust-region methods represent a well-established framework for multifidelity optimization [18,34]. In this paradigm, low-fidelity models serve as surrogates within a restricted trust region, the size of which is adapted based on the agreement between the predictions. This approach ensures global convergence through first-order consistency requirements: the low-fidelity model is corrected via additive or multiplicative adjustments to match both the objective value and gradient of the high-fidelity model at the trust region center [18,19]. Proposals are accepted or rejected based on the ratio of actual to predicted objective reduction. The trust region grows with good agreement, shrinks with poor agreement, and remains unchanged otherwise [19]. Recent extensions have incorporated reduced-order models within trust-region frameworks, adapting the reduced basis as the optimization progresses [35].

Surrogate-based optimization employs similar model management principles through infill criteria, such as expected improvement, which balances the exploitation of promising regions with the exploration of uncertain areas. These methods construct metamodels (e.g., kriging, radial basis functions) from expensive function evaluations, then use acquisition functions to select new sample points that are verified with the true objective [31,36].

In topology optimization, multifidelity methods have been explored using data-driven approaches employing deep generative models. Yaji et al. [37] introduced a framework that iteratively generates low-fidelity design candidates using variational autoencoders, then evaluates their performance on high-fidelity models. In further studies, this evolutionary algorithm-inspired method was incorporated with crossover-like operations via an autoencoder and mutation through constrained low-fidelity optimization [38]. Kawabe et al. [39] extended this concept with multi-channel variational autoencoders that simultaneously optimize material distributions and physical parameters. These approaches demonstrate that gradient-free multifidelity methods can address problems with strong nonlinearity.

Despite these advances, a notable gap exists in the topology optimization literature regarding explicit runtime verification with accept/reject mechanisms. Unlike trust-region methods in general optimization—where proposals are accepted or rejected based on measured agreement with high-fidelity evaluations [18,19]—existing multifidelity topology optimization frameworks typically rely on implicit fidelity management through iterative dataset refinement. VAE-based approaches update training datasets using high-fidelity analyses. They, however, lack proposal-by-proposal acceptance/rejection mechanisms that verify each coarse design on a fine mesh before inclusion [37,39]. This distinguishes current multifidelity TO methods from their counterparts in uncertainty quantification and general optimization, where acceptance-based filtering is standard [19].

### 2.3. Benchmark Context and Data-Driven Methods

**Figure 1:**
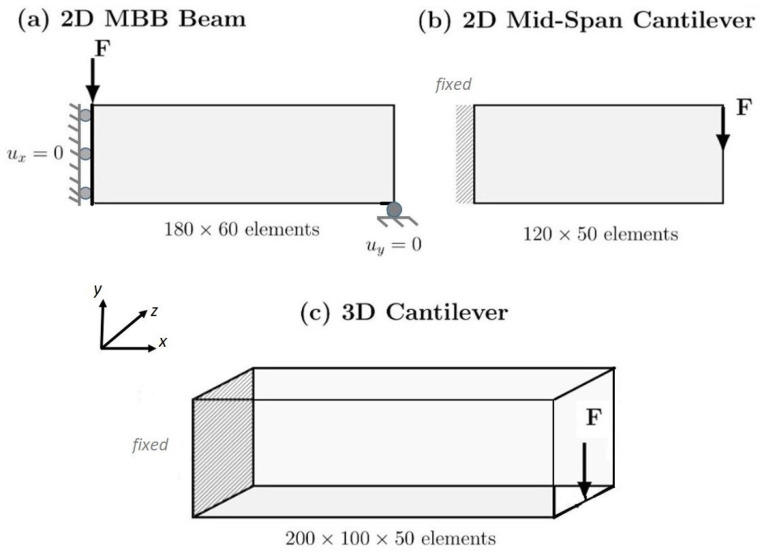
Benchmark problems and boundary/loading conditions used in this study.

The traditional benchmark problems used in this work—namely the MBB beam, mid cantilever, and 3D cantilever beam—are among the most popularly used in the topology optimization literature [12,17,40]. Figure 1 illustrates the geometry, boundary conditions, and loading for each problem in this study. In addition to serving as validation cases for traditional density-based methods, they have also served as training sets and test datasets for data-driven/machine learning (ML) approaches to topology optimization. Many recent data-driven approaches in TO literature use neural networks to predict optimized topologies directly from problem definitions [1,2,41,42]. Variational autoencoders and generative adversarial networks have been used to develop latent representations of topology spaces [37,39]. Physics-informed neural networks (PINNs) have been proposed to accelerate forward analysis during the optimization process [43,44,45]. Although some machine learning methods demonstrate impressive acceleration performance, comparing these systems is difficult because of the wide variety of architectures, training methodologies, and evaluation methods used [1,2].

### 2.4. Research Gaps

The literature reveals two gaps. First, although trust-region and surrogate-based methods employ explicit accept/reject verification [18,19,36], such safeguards are rarely explored in multifidelity topology optimization [38,39]. Second, systematic studies quantifying coarse-mesh proposal quality, acceptance rates, and cost–accuracy trade-offs in density-based TO remain limited [19,37].

## 3. Methodology and Experimental Design

### 3.1. Problem Formulation

The topology optimization problem has been formulated as a minimum compliance design under a volume constraint, based on the density-based SIMP approach [5]. All analyses assume linear elastic material behavior and small deformations. The design domain is discretized into finite elements, with each element assigned a continuous density variable $\rho_{e}\in \left[0,1\right]$, where $\rho_{e}=0$ represents a void and $\rho_{e}=1$ represents a solid material. The element’s Young’s modulus is interpolated as

(1) $$E\left(\rho_{e}\right)=E_{\min}+\rho_{e}^{p}\left(E_{0}-E_{\min}\right),$$

where $E_{0}$ is the Young’s modulus of the solid material, $E_{\min}$ is a small positive value that prevents numerical singularities, and *p* is the penalization factor that is typically set to 3 for compliance problems [3]. The optimization problem minimizes the structural compliance $C=u^{T}Ku$, where u is the global displacement vector and K is the global stiffness matrix, subject to equilibrium constraints Ku=F and a volume constraint $\sum_{e}v_{e}\rho_{e}\leq v_{f}V_{0}$, where $v_{e}$ is the element volume, $v_{f}$ is the prescribed volume fraction, and $V_{0}$ is the total design domain volume. A standard compact statement of the problem is [5]:

(2) $$\begin{array}{cc} \min_{\rho }\; & C\left(\rho \right)=u^{T}K\left(\rho \right)u, \end{array}$$

(3) $$\begin{array}{cc} s.t.\; & K(\rho )u=F, \end{array}$$

(4) $$\begin{array}{cc} \; & \sum_{e}v_{e}\rho_{e}\leq v_{f}V_{0}, \end{array}$$

(5) $$\begin{array}{cc} \; & 0\leq \rho_{e}\leq 1\;\forall e. \end{array}$$

Unless otherwise specified, all results presented in this study were performed in a nondimensionalized setting typically found in SIMP benchmarking studies [5,12,41,46]. Therefore, the element length is selected as the unit length; the solid stiffness is normalized to $E_{0}=1$ and the load magnitude is scaled with respect to its size. The calculated dimensionless compliance values can thus be interpreted as relative compliance values across different methods (all methods were run on a fine mesh). If compliance is required to be interpreted as an absolute quantity and to apply physical units, then compliance will have work units (i.e., work = N·m), where appropriate units of force and length have been defined.

To suppress checkerboard patterns and mesh-dependency artifacts, a density filter is applied [16]. The filtered density ${\tilde{\rho }}_{e}$ used in the finite element analysis is computed as the normalized distance-weighted average of ρ over a radius-$r_{\min}$ neighborhood, following Andreassen et al. [5]. The optimality criteria (OC) method is employed to update the design variables based on sensitivity analysis, following standard conventions in the topology optimization literature [5,13]. Convergence is monitored by tracking the change in design variables over a specified range of iterations. Optimization terminates when this change falls below a prescribed tolerance or when a maximum iteration limit is reached [15]. In multifidelity settings, geometric regularization is enforced by selecting the density filter radius in element units on each mesh to preserve a comparable minimum length scale in the physical space, and the exact values are benchmark-dependent.

### 3.2. Multifidelity Framework with Runtime Verification and Acceptance Control

The MFAS framework has been used to address the “trust” issue in coarse-mesh optimization by providing explicit runtime verification and acceptance control. Four method variants are compared. M1 operates on the fine mesh only (reference). M2 optimizes on the coarse mesh and evaluates the final design on the fine mesh at termination. M3 periodically verifies the design on the fine mesh every $f_{\mathrm{verify}}$ coarse iterations, with rollback on rejection. M4 extends M3 with a lower verification frequency and a fine-mesh cleanup phase starting from the best verified design. The combination of coarse-mesh exploration, fine-mesh verification with rollback to the best verified state, and fine-mesh cleanup from an independently verified starting point collectively mitigates stagnation in discretization-induced local optima.

The mechanism in M3 and M4 works as follows: the optimizer advances on the coarse mesh for a number of iterations defined by the verification frequency parameter $f_{\mathrm{verify}}$. At every verification checkpoint, the current coarse design $\rho_{c}$ is mapped onto the fine mesh via prolongation and bilinear upsampling (for 2D) or trilinear interpolation (for 3D), followed by filtering to obtain ${\tilde{\rho }}_{f}$, and evaluated via finite element analysis to obtain the compliance of the design on the fine mesh $C_{f}$. This compliance is compared to the best compliance obtained during optimization, $C_{\mathrm{best}}$. Table 1 summarizes the method variants (M1–M4), verification frequency, acceptance rules, and primary cost components.

In the 2D problem considered in this study, acceptance is based on the best compliance improvement margin and the ratio of actual to predicted compliance reduction [47]. This acceptance criterion compares two ratios of the compliance values:

(6) $$\eta =\frac{\Delta C_{\mathrm{actual}}}{\Delta C_{\mathrm{predicted}}}=\frac{C_{\mathrm{prev}}-C_{\mathrm{trial}}}{C_{\mathrm{coarse},\mathrm{old}}-C_{\mathrm{coarse},\mathrm{trial}}}.$$

Here, $C_{\mathrm{trial}}$ is the fine-mesh compliance of the prolonged trial design, $C_{\mathrm{prev}}$ is the fine-mesh compliance of the previously accepted state, and $C_{\mathrm{best}}$ is the best (lowest) verified fine-mesh compliance observed up to the current iteration. $C_{\mathrm{coarse},\mathrm{old}}$ and $C_{\mathrm{coarse},\mathrm{trial}}$ denote the corresponding coarse mesh compliances used to form the predicted reduction. The improvement margin is $\sigma =\sigma_{\mathrm{ratio}}\left|C_{\mathrm{best}}\right|$ and the trust ratio is compared against $\eta_{\mathrm{tr}}$. Both ratios must satisfy their respective conditions for acceptance:

1. $C_{f}\leq C_{\mathrm{best}}-\sigma$, where $\sigma =\sigma_{\mathrm{ratio}}\cdot \left|C_{\mathrm{best}}\right|$, and $\sigma_{\mathrm{ratio}}=0.001$;
2. $\eta \geq \eta_{\mathrm{tr}}$, where $\eta_{\mathrm{tr}}=0.10$.

If both conditions are satisfied, the design is accepted, and the next design is generated. If either condition is not met, the optimizer has the option to perform a limited number of “rollback” iterations with a decreasing step size until a new design is generated that satisfies both conditions [47]. In the 3D problem considered in this study, the acceptance criterion is based solely on the relative tolerance of the compliance of the design on the fine mesh to the best compliance value achieved up to the current iteration [19]:

(7) $$C_{f}\leq \left(1+\varepsilon_{\mathrm{rel}}\right)\;C_{\mathrm{best}},$$

where $\varepsilon_{\mathrm{rel}}=0.005$. Table 2 summarizes the MFAS acceptance parameters used in the 2D and 3D implementations. The acceptance parameters follow established conventions: the trust-ratio threshold $\eta_{tr}=0.10$ is within the standard range (0.1–0.25) used in trust-region optimization [18,34,47]; the SIMP parameters (p=3, OC move limit =0.2, filter radii $r_{\min}$) follow benchmarking standards [5,13,16]; verification frequencies and cleanup iterations were selected heuristically to investigate cost–quality trade-offs.

Upon successful verification, the fine-mesh evaluation becomes the new optimizer state, the coarse mesh is restricted from the accepted configuration, and $C_{\mathrm{best}}$ is updated if $C_{f}<C_{\mathrm{best}}$. When a design is rejected, the optimizer returns to the previous best verified design and continues from there. The configurations for the M3 and M4 method variants are as follows:

- for M3: Verification frequency $f_{\mathrm{verify}}=5$ for both 2D and 3D; no cleanup phase.
- for M4: Verification frequency $f_{\mathrm{verify}}=10$ for both 2D and 3D; includes cleanup phase with $N_{\mathrm{cleanup}}=25$ iterations (2D) or $N_{\mathrm{cleanup}}=15$ iterations (3D).

After coarse-guided optimization with verification has terminated (either by iteration limit or convergence), M4 initiates a fine mesh only optimization from the best verified design $\rho_{\mathrm{best}}$ and performs a maximum of $N_{\mathrm{cleanup}}$ SIMP iterations on the fine mesh. For the 2D version, the cleanup phase uses the OC move limit that is used in the standard SIMP iterations unless explicitly changed. The cleanup phase of the 3D version uses a reduced move limit to further improve the topological details by using the finer gradient meshes. This additional cleanup may result in improved solution quality compared to verification alone, but still benefits from the computational advantage of coarse mesh exploration.

### 3.3. Computational Environment

The 2D simulations were written in Python 3.12, and NumPy 2.0 and SciPy 1.16 were used for array operations and sparse linear algebra, respectively. The finite element analysis utilized four-node quadrilateral elements (Q4) and a standard isoparametric formulation [5]. The global stiffness matrix assembly is performed using vectorized NumPy operations to reduce the computational time [13]. Filtering is performed using precomputed sparse filtering matrices that are built once prior to optimization. Therefore, the filter can be applied efficiently at every iteration using sparse matrix-vector multiplication. The 2D experiments were performed on a single Intel Xeon CPU @ 2.20 GHz with 13.6 GB of RAM.

The 3D numerical experiments were developed in PyTorch 2.9 to utilize the GPU for the large computational sizes involved. The experiments were conducted using eight-node hexahedral (H8) elements with PyTorch tensors for all array operations [48]. PyTorch operations were utilized to perform vectorization of the finite element assembly, filtering, and sensitivity calculations where possible [49]. The linear systems were solved using PyTorch’s implementation of the preconditioned conjugate gradient (PCG) algorithm with Jacobi preconditioning [14]. If the PCG reaches the iteration cap or the relative residual remains above a threshold, a fallback re-solve is triggered with a looser tolerance and an increased iteration budget (Table 3) to obtain a valid displacement field for compliance evaluation. The iterative nature of this solver approach renders it suitable for parallelization on a GPU and scalable to the million+ degrees of freedom found in the 3D cantilever problem. The 3D experiments were run on an NVIDIA A100-SXM4-40GB GPU, which has 40 GB of memory, and an Intel Xeon processor running at 2.20 GHz, along with 89.6 GB of system RAM. GPU computation significantly reduces wall-clock time for 3D problems while maintaining double precision (float64). Table 3 lists the solver configuration used for the 3D experiments.

The wall-clock time included the initialization, loop iterations, filtering operations, finite element solves, sensitivity analysis, design variable updates, and verification checks. All methods used the same convergence rule based on the largest absolute change in design variables over the last $k_{\mathrm{consec}}$ iterations (2D: $k_{\mathrm{consec}}=8$, ${\mathrm{tol}}_{\mathrm{change}}=10^{-3}$; 3D: $k_{\mathrm{consec}}=5$, ${\mathrm{tol}}_{\mathrm{change}}=0.01$); however, the maximum iteration cap in 3D is method-dependent (M1: 300, M2: 100, M3: 200, M4: 200). To assess the statistical differences among the experimental results, paired comparisons were used for each combination of the test problem and random seed [21]. The performance of each optimization method can be compared while accounting for the influence of both problem-specific and seed-specific variability. The reported metrics include final verified compliance (fine-mesh), wall-clock time, speedup relative to M1, acceptance rate, and gray fraction.

### 3.4. Numerical Experiments

The framework was tested on three canonical benchmark problems in structural topology optimization: a 2D MBB beam, a 2D short cantilever (also referred to as mid-cantilever in the literature), and a 3D cantilever [5,13,15,37]. Table 4 lists the benchmark problem specifications, including the domain size, boundary conditions, loading, volume fraction, mesh, and filter radius.

Table 5 summarizes the optimization parameters used in the 2D and 3D experiments. Each benchmark problem was optimized using four methods: M1 (fine-mesh baseline), M2 (coarse mesh with final fine evaluation), M3 (MFAS with periodic verification at frequency $f_{\mathrm{verify}}=5$), and M4 (MFAS with verification at frequency $f_{\mathrm{verify}}=10$ and fine-mesh cleanup phase). The coarse mesh dimensions were scaled by a factor of 0.5 in each direction; the density filter radii $r_{\min}$ were specified per benchmark and per mesh (in element-length units) and are reported explicitly in Table 4. Each method–problem combination was repeated across five deterministic random seeds, introducing load perturbations (2D: shifting the loaded node in *y* by up to ±2 elements and applying a deterministic ±2% load magnitude jitter; 3D: shifting the loaded *z*-segment by up to ±2 elements), yielding 60 total optimization runs. The initial designs started from a uniform density field $\rho_{e}=v_{f}$. Table 6 shows the experimental campaign employing a full-factorial design with 60 optimization runs.

The performance metrics include the final verified compliance $C_{\mathrm{best}}$ (evaluated on a fine mesh), total wall-clock runtime, speedup relative to M1, acceptance rate (fraction of verification checks passing), and gray fraction (proportion of intermediate-density elements, $0.02<\rho_{e}<0.98$ for 2D or $0.05<\rho_{e}<0.95$ for 3D). The results were analyzed using paired statistical tests to assess the significance of the observed differences.

## 4. Results and Discussion

This section describes the results of 60 paired numerical optimization experiments performed for three different benchmark problems and four different methods using five deterministic seeds. All compliance values were computed using a fine mesh to ensure fairness when comparing the methods.

### 4.1. 2D Benchmark Problems

**Figure 2:**
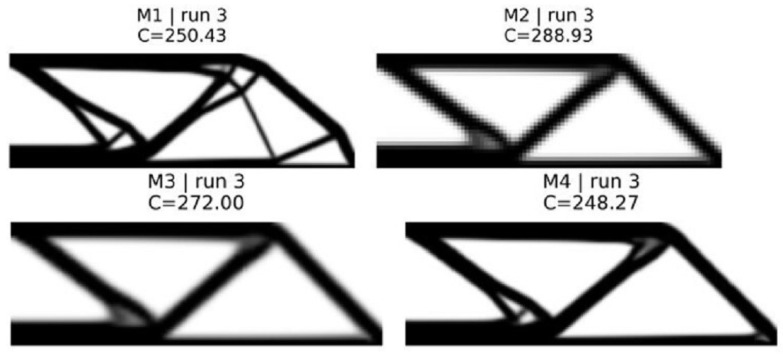
Final topologies for 2D MBB beam corresponding to the median seed.

**Figure 3:**
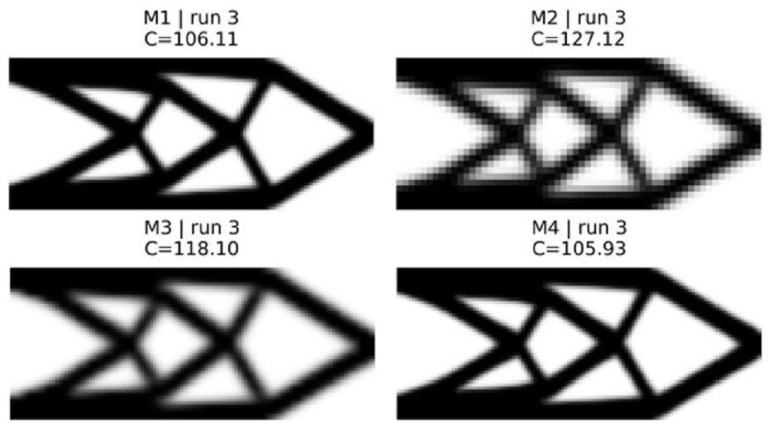
Final topologies for 2D cantilever beam corresponding to the median seed.

The results obtained for the MBB beam (180×60 fine mesh, $v_{f}=0.40$) and short cantilever (120×50 fine mesh, $v_{f}=0.50$) are shown in Figure 2 and Figure 3 and summarized in Table 7.

Because method M1 is based on SIMP benchmarks, it produces the best results with defined geometries and low gray values (≈0.26–0.31) [4,5]. Method M2 illustrates the basic “trust problem” for the first time: a coarse mesh reduces the computation time by a factor of six to seven compared with a fine mesh, but also leads to an increase in compliance of about +15 to +20%. Additionally, method M2 uses high gray values (0.45–0.65) and shows a diffuse distribution of material, which indicates that the discretization error has a significant influence on the optimizer, leading it into less optimal regions [19].

**Figure 4:**
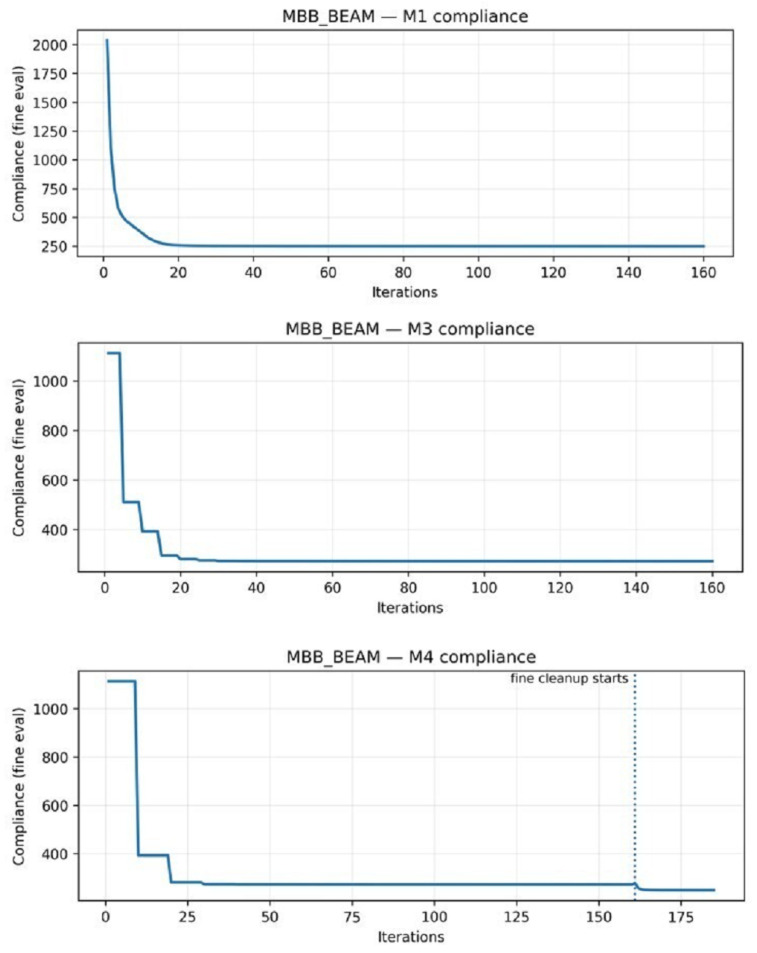
2D convergence history demonstrating verification behavior for representative 2D beam.

Method M3 partially recovers the lost quality by verifying periodically every five coarse iterations. The convergence history of both methods exhibits typical sawtooth patterns, in which the compliance increases after a rollback and decreases during the accepted verification steps (Figure 4). The MBB beam shows a conservative acceptance rate of 27% versus 41% for the cantilever, suggesting that load-path complexity affects the coarse–fine discrepancy. Nevertheless, coarse–fine optimization using method M3 can achieve a speed-up of only 1.25–1.34× due to the fact that about 33 verification checks consume the largest part of the overall computational costs [18].

Method M4 addresses this issue by reducing the frequency of verification ($f_{\mathrm{verify}}=10$) and introducing a cleanup phase of 25 iterations. Method M4 achieves near-equivalent compliance to method M1 (compliance differences of −0.84% to −0.18%, statistically significant per paired *t*-test with $p<10^{-7}$ but practically negligible). At the same time, method M4 achieves a meaningful speed-up (1.43–1.52×). The cleanup phase eliminates intermediate density artifacts, producing gray values comparable to or lower than method M1. Strategic workload allocation—coarse exploration, infrequent verification, and targeted cleanup—achieves this cost–quality balance.

### 4.2. 3D Cantilever Problem

**Figure 5:**
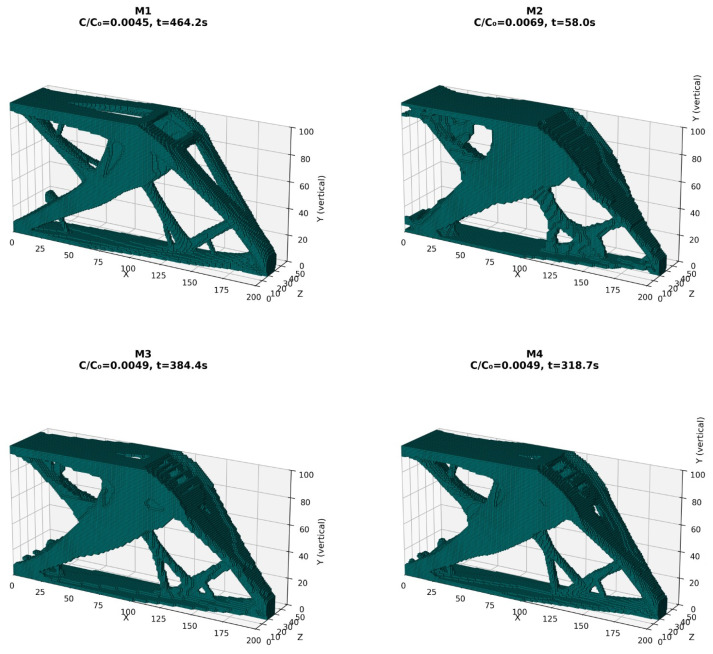
3D cantilever final topologies (median seed across methods). Visualization threshold τ=0.3; Each panel reports the normalized compliance $C/C_{0}$ and wall-clock time *t* (s), where $C_{0}$ is the compliance of the corresponding initial/reference design used for normalization in the implementation.

The 3D cantilever (200×100×50 fine mesh, $v_{f}=0.10$) allows the demonstration of the method behavior at realistic scales with more than three million degrees of freedom (DOF). The compliance loss of method M2 (+56.5%) in the 3D case is much worse than in the two-dimensional cases, because the coarse mesh introduces a larger model discrepancy in three dimensions [13,14]. The deterioration of the compliance loss with increasing problem complexity confirms that coarse-mesh optimization alone is not suitable for producing 3D applications (Figure 5).

In the 3D problem, methods M3 and M4 used a relative acceptance criterion

$$C_{f}\leq \left(1+\varepsilon_{\mathrm{rel}}\right)\;C_{\mathrm{best}},\;\varepsilon_{\mathrm{rel}}=0.005,$$

instead of the dual acceptance criterion used in the 2D model. This simpler formulation is more liberal and yields acceptance rates of 93% (M3) and 92% (M4) for the 3D cantilever, which are significantly higher than those of the 2D benchmarks. Owing to their high acceptance rates, methods M3 and M4 can make continuous progress along coarse meshes. However, Method M3 shows a large variability in runtime (CV=37.35%), which is caused by the varying number of iterations needed to trigger the early stop, depending on the random seed used. Method M4 retains its stable behavior (CV=18.24%), thanks to the reduced verification frequency and a fixed-duration cleanup phase, and delivers a speed-up of 1.65× with a compliance loss of +8.3% (Table 8).

### 4.3. Discussion

The cross-problem analysis (Table 9) leads to the following main findings. First, coarse-mesh errors are problem-dependent: M2’s compliance loss ranges from +15% (2D) to +57% (3D), so multifidelity methods must account for both dimensionality and geometric complexity. Second, verification frequency involves a trade-off: frequent verification (M3) recovers quality but limits speedup, while less frequent verification (M4) exploits coarse–fine alignment when acceptance criteria are well calibrated. Third, gray fraction correlates strongly with compliance and serves as a fast quality indicator.

The MFAS is most beneficial when the fine-mesh optimization dominates the total runtime (e.g., several hundred seconds per optimization run, as observed in 3D problems), coarse–fine alignments are moderate (acceptance rates >30%), and the application tolerates small compliance losses (<10%) during the design exploration process. Unlike machine learning-based methods [2,37,39,41,50] that require problem-specific training sets, the MFAS provides immediate acceleration of the optimization without any offline phases. The framework is currently limited to single-objective linear elastic compliance minimization. Extensions to nonlinear [51,52], multi-objective [7,53,54], or multiphysics problems [55,56] require reformulation of the acceptance criterion and might face increased coarse–fine model discrepancy. The fixed 2× coarse-to-fine mesh ratio does not adapt to local feature complexity, contributing to problem-dependent acceptance rates. Therefore, it is positioned as a practical tool for production workflows, where rapid design iterations take priority over absolute optimality.

## 5. Conclusions

In this study, an acceptance safeguard framework for multifidelity density-based topology optimization with explicit runtime verification and acceptance control is developed. Experimental results indicate that MFAS with fine mesh cleanup produces speed-ups of 1.4–1.7× over fine mesh baseline optimizations; the fine evaluated compliance gap was between −1% and +8% relative to fine mesh reference solutions; and no offline training or surrogate development were used. Additionally, the framework is easy to implement for workflows in which reduced wall-clock time and rapid design iteration are important while preserving bounded degradation in compliance.

The principal contribution is a systematic framework that integrates periodic fine mesh verification, the best referenced acceptance criteria, and targeted cleanup to balance solution reliability with computational efficiency. Statistical analysis of 60 paired optimization runs demonstrated that the coarse mesh discretization error is benchmark dependent and can be significant; +15% to +20% in 2D, versus +57% in 3D for the coarse-only method. These results support the need for multifidelity approaches to consider both the dimensionality of the problem and the potential for coarse–fine model mismatch. Verification scheduling represents a trade-off between solution quality and speed-up: conservative verification schedules (i.e., every 5 coarse iterations) will recover some lost quality but may severely limit the acceleration available. Aggressive schedules (every 10 iterations) with cleanup can exploit coarse–fine alignment when properly calibrated.

Methodological improvements in future research include adaptive verification schedules based on acceptance rates, extending the hierarchical nature of the approach to include multiple levels of fidelity, and incorporating data-driven surrogate models into the MFAS safety mechanisms. Future applications of the MFAS framework could include multiphysics topology optimization, manufacturing constraint integration (e.g., additive manufacturing overhang restrictions), multimaterial designs, and scalability studies. The MFAS framework provides a reliable multifidelity optimization environment in situations where rapid iterative design with explicit runtime verification is emphasized.

## Figures and Tables

**Table 1:** Comparison of method variants.

Method	Description	Verification Frequency	Acceptance Rule	Primary Cost Component
M1	Fine-mesh baseline	–	–	Fine FEA only
M2	Coarse-only + final evaluation	–	–	Coarse FEA + fine evaluation at termination
M3	MFAS with frequent verification	$f_{\mathrm{verify}}=5$	Dual (2D)/ relative (3D)	Coarse FEA + periodic fine verification
M4	MFAS with cleanup	$f_{\mathrm{verify}}=10$	Dual (2D)/ relative (3D)	Coarse FEA + verification + fine cleanup

Remarks: Primary cost components refer to computational workload distribution.

**Table 2:** MFAS acceptance parameters.

Parameter (Symbol)	2D Value	3D Value	Applicable Methods
Trust-region threshold ($\eta_{\mathrm{tr}}$)	0.10	–	M3, M4 (2D only)
Improvement margin ratio ($\sigma_{\mathrm{ratio}}$)	0.001	–	M3, M4 (2D only)
Backtracking attempts	2	–	M3, M4 (2D only)
Step shrink factor	0.5	–	M3, M4 (2D only)
Relative tolerance ($\varepsilon_{\mathrm{rel}}$)	–	0.005	M3, M4 (3D only)

Remarks: All threshold and ratio parameters are dimensionless quantities. Backtracking attempts is an iteration count.

**Table 3:** Solver configuration for 3D experiments.

Parameter	Value	Description
PCG tolerance	$10^{-6}$	Convergence criterion
PCG max iterations	400	Iteration limit
PCG fallback tolerance	$10^{-4}$	Used if the initial PCG solve fails
PCG fallback max iterations	1200	Increased iteration limit in fallback re-solve
Precision	float64	Double precision
Preconditioning	Jacobi	Preconditioner type

Remarks: PCG tolerances are dimensionless relative residual norms.

**Table 4:** Benchmark problem specifications.

Field	2D MBB Beam	2D Mid-Cantilever	3D Cantilever
Domain size (elements)	180×60	120×50	200×100×50
Boundary conditions	Left edge: $u_{x}=0$; Bottom-right: $u_{y}=0$	Left edge: fixed	Left face: fixed
Load	Vertical point load, top-left corner	Vertical point load, right midpoint	Vertical patch load, bottom edge
Volume fraction ($v_{f}$)	0.40	0.50	0.10
Filter radius (fine) ($r_{\min,f}$)	1.5	1.5	2.0
Filter radius (coarse) ($r_{\min,c}$)	1.5	1.5	1.0

Remarks: 2D MBB beam is modeled as half-MBB with symmetry at x = 0; for 3D Cantilever, load is applied as downward line load over a short z-segment (3 nodes); filter radii $r_{\min}$ are specified in element-length units. Load magnitudes are normalized to unity under the nondimensionalized convention stated in Section 3.1.

**Table 5:** Optimization parameters used in the study.

Parameter (Symbol)	2D Value	3D Value	Description
Penalization (*p*)	3	3	SIMP interpolation exponent
Young’s modulus ($E_{0}$)	1.0	1.0	Solid material stiffness
Void stiffness ($E_{\min}$)	$10^{-3}$	$10^{-6}$	Regularization parameter
Poisson’s ratio (ν)	0.3	0.3	Material property
OC move limit	0.2	0.2	Design update bound
Convergence tolerance (${\mathrm{tol}}_{\mathrm{change}}$)	$10^{-3}$	0.01	Density change threshold
Consecutive iterations ($k_{\mathrm{consec}}$)	8	5	Convergence criterion
Maximum iterations	160	200–300	Problem-dependent
Gray threshold ($t_{\mathrm{gray}}$)	0.02	0.05	Intermediate density range
Random seeds	5	5	Statistical replications
Load perturbation	±2 elements	±2 elements	Seed-dependent jitter

Remarks: All parameters follow the nondimensionalized convention of Section 3.1 with $E_{0}=1$ and unit normalized loading; $E_{\min}$ is reported as a dimensionless fraction of $E_{0}$.

**Table 6:** Experimental design: full factorial structure (3×4×5=60 runs).

Factor	Levels	Values	Runs per Factor
Problems	3	2D MBB bBeam, 2D Mid-cantilever, 3D cantilever	3×20=60
Methods	4	M1 (fine), M2 (coarse), M3 (MFAS: $f_{\mathrm{verify}}=5$), M4 (MFAS: $f_{\mathrm{verify}}=10$)	4×15=60
Seeds	5	0, 1, 2, 3, 4	5×12=60
Total Optimization Runs			60

Paired design: Same seed applied across all methods within each problem. Seed perturbations: 2D load node shift in *y* by ±2 elements + load magnitude jitter ±2% of normalized unit load; 3D load *z*-segment offset ±2 elements. Statistical analysis: Paired *t*-tests (controls problem and seed effects).

**Table 7:** Summary of 2D benchmark problems (mean ± std across 5 seeds).

Problem	Method	*C*	Gray	Time (s)	Speedup	Accept
MBB Beam (180 × 60, $v_{f}=0.40$)	M1	250.02 ± 0.62	0.264 ± 0.000	73.0 ± 7.5	–	–
	M2	288.54 ± 0.54	0.456 ± 0.000	11.5 ± 0.4	6.34	–
	M3	271.77 ± 0.48	0.345 ± 0.000	58.6 ± 0.7	1.25	0.27
	M4	247.92 ± 0.58	0.203 ± 0.000	50.8 ± 0.5	1.43	0.34
Short Cantilever (120 × 50, $v_{f}=0.50$)	M1	106.14 ± 0.04	0.302 ± 0.001	37.0 ± 0.5	–	–
	M2	127.07 ± 0.06	0.654 ± 0.000	5.8 ± 0.7	6.47	–
	M3	118.13 ± 0.09	0.535 ± 0.002	27.7 ± 0.4	1.34	0.41
	M4	105.95 ± 0.05	0.298 ± 0.001	24.4 ± 0.5	1.52	0.55

Remarks: Reported compliance *C* is dimensionless under the nondimensionalization of Section 3.1 and is always evaluated on the fine mesh for all methods (including coarse-only runs); gray = gray fraction (proportion of elements with $0.02<\rho_{e}<0.98$); time = wall-clock runtime; speedup = mean speedup relative to M1; accept = verification acceptance rate (M3, M4 only). Coarse mesh sizes: MBB 90 × 30, Cantilever 60 × 25. Filter radii: $r_{\min,f}=1.5$ (fine), $r_{\min,c}=1.5$ (coarse). Penalization factor p=3 for all methods.

**Table 8:** Summary of 3D cantilever problem (mean ± std across 5 seeds).

Method	*C*	ΔC vs. M1 (%)	Time (s)	Speedup	Gray	Accept	FEA ^†^
M1	4.48 ± 0.04	0.0 ± 0.0	465.1 ± 23.8	1.00	0.078 ± 0.004	–	255
M2	7.01 ± 0.24	+56.5 ± 4.8	57.0 ± 0.6	8.16	0.101 ± 0.003	–	12
M3	4.97 ± 0.12	+10.9 ± 2.2	328.5 ± 122.7	1.86	0.089 ± 0.004	0.93 ± 0.16	58
M4	4.85 ± 0.04	+8.3 ± 1.4	294.5 ± 53.7	1.65	0.098 ± 0.003	0.92 ± 0.18	56

Remarks: Fine mesh: 200 × 100 × 50 elements, $v_{f}=0.10$. Coarse mesh: 100 × 50 × 25 elements. Filter radii: $r_{\min,f}=2.0$ (fine), $r_{\min,c}=1.0$ (coarse). *C* = final compliance (dimensionless, fine-mesh evaluated); ΔC = compliance gap; time = wall-clock runtime; speedup = mean speedup relative to M1; gray = gray fraction (proportion with $0.05<\rho_{e}<0.95$); accept = verification acceptance rate; ^†^ FEA = fine-equivalent FEA solves (coarse solves scaled by ≈0.125). Penalization factor p=3 for all methods. M3 and M4 use relative acceptance criterion with $\varepsilon_{\mathrm{rel}}=0.005$.

**Table 9:** Paired statistical comparisons against M1 baseline.

Problem	Method	*C*	$t_{C}$	$p_{C}$	Time (s)	$t_{\mathrm{time}}$	$p_{\mathrm{time}}$
MBB Beam	M1	250.02 ± 0.62	–	–	73.0 ± 7.5	–	–
	M2	288.54 ± 0.54	997.63	$6.06\times 10^{-12}$	11.5 ± 0.4	−19.04	$4.48\times 10^{-5}$
	M3	271.77 ± 0.48	313.00	$6.25\times 10^{-10}$	58.6 ± 0.7	−4.37	$1.20\times 10^{-2}$
	M4	247.92 ± 0.58	−109.09	$4.23\times 10^{-8}$	50.8 ± 0.5	−6.68	$2.61\times 10^{-3}$
Short Cantilever	M1	106.14 ± 0.04	–	–	37.0 ± 0.5	–	–
	M2	127.07 ± 0.06	1090.93	$4.24\times 10^{-12}$	5.8 ± 0.7	−59.09	$4.91\times 10^{-7}$
	M3	118.13 ± 0.09	353.72	$3.83\times 10^{-10}$	27.7 ± 0.4	−27.99	$9.70\times 10^{-6}$
	M4	105.95 ± 0.05	−51.20	$8.71\times 10^{-7}$	24.4 ± 0.5	−29.85	$7.51\times 10^{-6}$
3D Cantilever	M1	4.48 ± 0.04	–	–	465.1 ± 23.8	–	–
	M2	7.01 ± 0.24	23.46	<0.0001	57.0 ± 0.6	−37.74	<0.0001
	M3	4.97 ± 0.12	9.81	$6.05\times 10^{-4}$	328.5 ± 122.7	−2.22	$9.08\times 10^{-2}$
	M4	4.85 ± 0.04	12.25	$2.55\times 10^{-4}$	294.5 ± 53.7	−5.44	$5.54\times 10^{-3}$

Remarks: Paired *t*-test results comparing methods M2, M3, and M4 against M1 baseline. Statistics are computed from seed-wise paired differences (paired design runs sharing the same random seed), with n=5 pairs per problem–method combination, controlling for seed effects. Compliance *C* is dimensionless (Section 3.1, fine-mesh evaluated for all methods); runtime is in seconds. The reported $t_{C}$ and $t_{\mathrm{time}}$ are paired *t*-statistics for compliance and runtime, respectively; corresponding *p*-values quantify statistical significance. Positive $t_{C}$ indicates higher compliance (worse quality); negative $t_{\mathrm{time}}$ indicates faster runtime (better efficiency). Note: Statistical *p*-values reported in this table are unrelated to the SIMP penalization exponent *p* used in the optimization formulation (Section 3.1).

## Data Availability

The original contributions presented in this study are included in the article. Further inquiries can be directed to the corresponding author.
